# Ureteroscopy and stone treatment in the elderly (≥70 years): prospective outcomes over 5- years with a review of literature

**DOI:** 10.1590/S1677-5538.IBJU.2017.0516

**Published:** 2018

**Authors:** Sarah Prattley, James Voss, Stephanie Cheung, Robert Geraghty, Patrick Jones, Bhaskar K. Somani

**Affiliations:** 1 University Hospital Southampton, NHS Trust, United Kingdom, UK

**Keywords:** Ureteroscopy, Calculi, Therapeutics

## Abstract

**Objective:**

To assess outcomes of ureteroscopy for treatment of stone disease in the elderly. Ureteroscopy (URS) is an increasingly popular treatment modality for urolithiasis and its applications are ever expanding with the development of newer technologies. Its feasibility and outcomes within the elderly population to our knowledge remain under-reported.

**Materials and Methods:**

We examined the patient demographics and surgical outcomes from our prospective database for patients ≥70 years who underwent URS for urolithiasis, in a 5-year period between March 2012 and December 2016.

**Results:**

A total of 110 consecutive patients underwent 121 procedures (1.1 procedure/patient) with a mean age of 77.2 years (range: 70-91 years). Stone location was in the kidney/ pelviureteric junction (PUJ) in 29%, ureter in 37% and in multiple locations in 34%. The initial and final stone free rate (SFR) was 88% and 97% respectively. While 73% were done as true day case procedures, 89% patients were discharged within 24 hours. Eleven patients (9%) underwent complications of which 10 were Clavien I/II including acute urinary retention, urinary tract infection, stent symptoms and pneumonia. One patient underwent Clavien IV complication where they needed intensive care unit admission for urosepsis but fully recovered and were discharged home subsequently.

**Conclusion:**

Ureteroscopy is a safe and effective method of managing urolithiasis in elderly patients. Although most patients are discharged within 24-hours, consideration needs to be made for patients where social circumstances can impact their discharge planning.

## INTRODUCTION

The elderly population worldwide is rising. In the United Kingdom (UK) those aged over 75 are set to nearly double from 5.2 million in the year 2014 to 9.9 million by 2039 (1). There is an increasing burden of urinary tract stone disease and a rising trend towards surgical management, of which ureteroscopy (URS) is the fastest growing intervention (2). The number of ureteroscopic stone treatments has increased by 252% between 1996 and 2016 (2). With the modern evolution and technological advancement in URS, it is now recommended as a first line treatment for intra-renal stones less than 1.5cm (3).

Stone formation in the elderly (>65 years) has been reported to be between 9.6-16% of all stone patients, with a lifetime prevalence of 14% (4-6). Although a rise in the incidence of urolithiasis was seen across all ages, this was highest in those over 75 years where it increased by 51% in a span of 7 years (2006/2007 to 2013/2014) (4). This is thought to be due to increasing life expectancy (6). However, given the differing metabolic profile, stone composition, and co-morbidity, urolithiasis in the elderly should not be viewed merely as an extension of the population of younger stone formers, but as a disease in its own right (7, 8).

The efficacy of surgical intervention for urolithiasis in the elderly has yet to be clarified due to a paucity of evidence and contradictory results (9). We report on the outcomes for a consecutive cohort of elderly patients who underwent ureteroscopy for treatment of their stone disease with a review of literature.

## MATERIALS AND METHODS

Prospective data collection for consecutive patients was undertaken over a 5-year period between March 2012 and December 2016, 703 patients underwent ureteroscopy for stone disease during this time. Of these patients, 110 (16%) were aged ≥70 years and underwent 121 procedures for stones in the kidney or ureter. Demographic and clinical variables were prospectively collected and are presented in Tables 1 and 2, respectively. The diagnosis of urolithiasis was confirmed by a non-contrast CT scan (CTKUB).

**Table 1:** Patient demographics and stone location (PUJ – pelvic-ureteric junction).

No. of patients (procedures)		110 (121)
Mean age, years (range)		77.2 years (70-91 years)
70-75		43
75-80		35
80-85		29
>85		14
ASA I/II/III/IV		7/63/50/1
Median age, years		77
Pre-operative creatinine (μmol/L), mean±SD		104±57
Pre-operative stent n(%)		32 (26%)
Pre-operative positive urine culture (appropriately treated pre-operatively) n (%)		32 (26%)
Multiple stones n (%)		40 (32%)
**Stone position (single)**		
	Upper pole, n (%)	2 (2%)
	Middle pole, n (%)	4 (3%)
	Lower pole, n (%)	16 (13%)
	PUJ / Renal pelvis, n (%)	13 (11%)
	Upper ureter, n (%)	6 (5%)
	Middle ureter, n (%)	15 (12%)
	Lower ureter, n (%)	24 (20%)
**Stone position (multiple)**		
	Multiple ureteric n(%)	15 (12%)
	Multiple Renal n(%)	16 (13%)
	Multiple ureteric and renal n(%)	9 (7%)
	Mean largest individual stone diameter (range)	10.6 (3-37)
	Mean cumulative stone diameter, mm (range)	17.1 (3-156)

**Table 2:** Operative details and patient outcomes.

Operative details		
Mean operative time (minutes)±SD		50±25
Use of an access sheath (%)		43 (36%)
Length of stay (days), mean (range)		2.1 (0-90)
Day case		88 (73%)
<24 hours		19 (16%)
1-3 days		5 (4%)
>3 days		8 (7%)
Post-operative drainage (JJ stent, ureteric catheter, stent on string), n (%)		118 (98%)
**Stone composition**		
	Calcium Oxalate	66 (64.1%)
	Calcium Phosphate	28 (27.2%)
	Magnesium Phosphate	5 (4.9%)
Uric Acid		4 (3.9%)
Singular Stone Composition		59 (65.6%)
Mixed Stone Composition		31 (34.4%)
Surgical complications, n(%)		11 (9%)
Acute urinary retention (Clavien I/II)		4 (3%)
Urosepsis (Clavien IV)		1 (1%)
Urinary tract infection (Clavien I/II)		4 (3%)
Post-operative stent pain (Clavien I/II)		1 (1%)
Pneumonia (Clavien I/II)		1 (1%)
Overnight stay for social reasons (frail, stay alone)		12 (10%)
Overnight stay for patients who underwent elective catheterisation		6 (5%)
Initial stone free rate (SFR)		97 (88%)
Final SFR		107 (97%)

All patients underwent ureteroscopy (URS) and stone fragmentation/retrieval under a general anaesthesia, with stones send for analysis when retrieved. A post-operative drainage with JJ stent or ureteric catheter or stent on a string was done in majority of patients. A urethral catheter was not routinely placed unless there was a history of previous urinary retention. A repeat URS was either planned due to a large initial stone burden or if they were symptomatic with residual stones on follow-up. A post-operative follow-up was done at 3 months with a plain KUB XR for radiopaque stones or ultrasound scan (USS) for radiolucent stones. Stone free rate (SFR) was defined as endoscopically or radiologically stone free or with fragments ≤2mm.

A review of literature on all articles reporting on URS for stone disease in elderly was also carried out (10-14).

## RESULTS

110 patients underwent 121 procedures (1.1 procedure/patient), with 11 patients undergoing repeat URS either as a planned staged procedure due to the initial stone burden or if they had symptomatic residual stones on follow-up (Table-1). The mean age of patients was 77 years (range: 70-91 years) with a male: female ratio of 3:1.

The stone location was in the kidney/pelvic ureteric junction (PUJ) in 29%, ureter in 37% and in multiple locations in 34%, with a mean stone size of 10.6mm (range 3-37mm) and the cumulative stone length was 17mm (range 3-156mm). Five patients (4%) underwent bilateral URS for stone disease. With a mean (±SD) operative time of 50±25 minutes, a ureteric access sheath (UAS) was used in 36% (43 procedures), and a post-operative drainage (JJ Stent, ureteric catheter or stent on string) was inserted in 98% (118 procedures). An elective urethral catheter was placed in 6 (5%) procedures.

The stone composition was predominantly calcium oxalate (64.1%), but also included calcium phosphate (27.2%), magnesium phosphate (4.9%) and uric acid (3.9%). A combination of stone composition was found in 34.4% of cases. Post-operatively, length of stay (LOS) was limited to day case surgery in 73% of cases (0-days), with 16% being discharged within 24 hours of the procedure (Table-2). A further 4% of patients were discharged between days 1-3, and 7% of patients required admission for >3 days, which was either due to post operative complications or social circumstances delaying discharge despite being medically fit.

The initial and final SFR was 88% (n=97) and 97% (n=107) respectively. The overall complication rate was 9% (n=11), ten patients with Clavien I/II complications and one with Clavien IV complication. Of the Clavien I/II complications, 4 developed acute urinary retention, 4 had a urinary tract infection and one patient each had stent discomfort and pneumonia. The patient with Clavien IV complication required admission to the Intensive Care Unit for management of E. coli urosepsis post operatively. He was managed appropriately with intravenous antibiotics and vasopressor support for refractory hypotension, before being stepped down to the ward and subsequently discharged home. All patients recovered and were discharged home or to their residential/nursing home.

### Ureteroscopy (URS) in the elderly (Literature review)

Our literature review shows a total of six studies (including our study) (Tables 3-5) that reports on the outcomes of ureteroscopy in elderly reporting a total of 560 patients with a complication rate of 12.3% (n=69) (Table-5) (10-14). Recent advancements in URS through endoscope miniaturisation, improved deflection techniques, enhanced optical quality, have lead to an increase in popularity of URS as a first line treatment for urolithiasis (3, 15, 16). There is a significant paucity of evidence in the use of URS for the treatment of urolithiasis in elderly patients with only few previous studies reported (Tables 3-5).

**Table 3:** Patient demographics across other studies reported in the literature.

Study	Type of study	Country of origin of study	Number of Patients (procedures)	Definition of elderly (years)	Mean Age, years ± SD (range)	Mean largest individual stone diameter, mm ± SD (range)	Mean cumulative stone diameter, mm ± SD (range)
Akman et al. 2012 (11)	Prospective	Turkey	28	>65	68.9±4.1	15 to 30 (no mean available)	/
Tolga-Gulpinar et al. 2015 (10)	Retrospective	Turkey	170	>60	66.5 (61-87)	/	17.2 (7.2)
Hu et al. 2016 (12)	Retrospective	China	80	>60	65.1±5.2	/	15.8 (3.4)
Yoshioka et al. 2016 (13) 65-74 Years >75 Years	Retrospective	Japan	42	65-74	69.26±2.92	9.56±3.27	/
			39	>75	79.46±4.69	8.80±3.16	/
Berardinelli et al. 2017 (14)	Prospective	Italy	91	>65	72.1±5.06	/	13.05 (5.79)
Current Study	Prospective	UK	110 (121)	>70	77.2 (70-91)	10.6 (3-37)	17.1 (3-156)

**Table 4:** Operative details in other studies reported in the literature.

Study		Mean Operative time, mins ± SD (range)	Access Sheath use (%)	LOS, mean days ± SD (range) median	Initial SFR (%)	Final SFR (%)	Complications, n (%)
Akman et al. 2012 (11)		64.5±20.9	N/A	1.1±0.44	82.10%	92.80%	2 (7.1%)
Tolga-Gulpinar et al. 2015 (10)		53±23.4	82.50%	1.6 (1-18)	N/A	81.10%	14 (7.6%)
Hu et al. 2016 (12)		75.9±34.0	100%	5.6±2.4	40.80%	65.80%	11 (13.75%)
Yoshioka et al. 2016 (13)							
	65-74 Years	72.43±30.51	N/A	N/A	N/A	78.57%	5 (11.9%)
	>75 Years	70.67±30.58	N/A	N/A	N/A	92.31%	5 (12.82%)
Berardinelli et al. 2017 (14)		64.31±31.87	82.40%	2.8±1.8	65.93%	N/A	9 (9.89%)
Current Study		50.0±25.0	36%	0 days (median)	88%	97%	11 (9%)

**Table 5:** Nature of complications across all studies.

Study		Overall Complications, n (%)	Complications
Akman et al. 2012 (11)		2 (7.1%)	Renal colic (n=2)
Tolga-Gulpinar et al. 2015 (10)		26 (15.1%)	Intraoperative surgical comp's (n=13); Perioperative medical comp's (n=2); Post-operative infection (n=11)
Hu et al. 2016 (12)		11 (13.7%)	Septic shock (n=1); N+V (n=1); Fever (n=9);
Yoshioka et al. 2016 (13)		5 (11.9%)	Post-operative pyelonephritis (n=5)
	65-74 Years		
	>75 Years	5 (12.8%)	Post-operative pyelonephritis (n=5)
Berardinelli et al. 2017 (14)		9 (9.9%)	Bleeding (n=2); Fever (n=3); Perforation of pelvis/calyx (n=2); ureteral injury (n=1); non-obstructive pyelonephritis (n=1)
Current Study		11 (9%)	Urosepsis (n=1); Acute urinary retention (n=4); UTI (n=4); Pain (n=1); Pneumonia (n=1)

## DISCUSSION

### Meaning and strengths of the study

With a rise in the incidence of stone disease in elderly and the use of URS for its treatment, we report on unselected consecutive elderly patients who underwent ureteroscopic management of their stone disease. Our data shows excellent SFR (97%) with low complication rates (9%, mainly Clavien I/II) for these patients where vast majority were done either as true day case procedures (73%) or were discharged within 24 hours (89%), without the routine use of urethral catheter post-operatively. While bilateral same session ureteroscopy was successfully done in all 5 patients, stones >1.5cm seem to need a second procedure due to a large stone burden.

Our study demonstrates comparable SFR and complication levels to those published in SWL and PCNL, and provides further evidence of the efficacy of URS in the management of elderly stone disease. We also sub-analysed the data on 43 patients (46 procedures) over the age of 80 years, and the SFR and complication rates were similar to the overall cohort.

### Comparison of all studies published in the literature

Tolga-Gulpinar et al. reported a SFR of 81.1% for patients >60 years; this was comparable to the non-matched groups being <15 years (SFR 78.4%) and 16 - 60 years (SFR 77.5%) (10). Multivariate logistic regression only found that stone size and number had an impact of SFR. This is comparable to our level of SFR following single URS being 88%. A similar study from Japan stratifying by age of patients showed that URS is the preferred treatment for elderly patients even for those with multiple comorbidities (13).

Complication rates for URS has been demonstrated to be less than that of a matched PCNL group, being 7.1% and 10.7% respectively, although not statistically significant (11). This is comparable to the intraoperative and perioperative complication rates found by Tolga-Gulpinar being 7.6% and 1.1% for the >60 year old group (10). We present a postoperative complication rate of 9% with the majority of these being either acute urinary retention or urinary tract infection. Only two patients suffered with urosepsis or pneumonia, and no mortality occurred in our series.

The attempted shift towards urolithiasis management in the outpatient setting remains achievable in the majority of geriatric patients, with a continued decrease in bed days required for management (15.8%) and rising day case procedures (9.7%) (12). Our results demonstrate that day-case URS was possible in 73% of patients, and a 24-hour discharge achievable in 89%, easing the demand for acute hospital admission and providing a cost-effective service. This is in concordance with other published LOS following URS in the elderly being 1.4 days on average for those >60 years (10).

### Troubleshooting for URS in elderly

Although URS can be successfully done in elderly patients with good outcomes, there can be certain difficulties encountered during the procedure (17). Enlarged prostate can pose difficulties in access to the ureteric orifice. Similarly, care should be taken to minimise the risk of anaesthetic complications and urosepsis by pre-operative optimisation of these patients. Although a post-operative catheter is not usually necessary and most patients can be done as a day-case procedure, this might be helpful in some patients (18). As the cost of performing a URS decreases, this might prove to be a more cost efficient treatment in elderly (19). It seems like the remit of URS in elderly is increasing and the trends are similar to those seen in obesity and paediatric patients (20, 21).

### Shockwave Lithotripsy (SWL) and percutaneous nephrolithotomy (PCNL) in the elderly

SWL is the least invasive method for the surgical management of urolithiasis, however it is not free of complications, normally related to the passage of stone fragments or residual stone fragments, which can cause an infection (22). There have been conflicting results on the impact age for the outcome of SWL relating to its SFR. The success of SWL has been attributed to the stone size, location, renal anatomy, stone composition and the type of lithotripter, however age, in a study by Al-Ansari, did not impact overall SFR (23). Conversely, in a study by Abdel-Khalek, which included 2954 patients with renal stones treated by SWL, demonstrated that, in multivariate analysis, age >40 years was a predictor of SWL failure (24).

SFR for the use of SWL in the elderly appears to vary widely from 37.6% - 87.1% (8, 9, 25-27). Siginholfi et al. reported on the outcomes for a cohort of 130 patients over the age of 70 who underwent shockwave lithotripsy for treatment of renal and ureteric stones. They found 52.1% of patients to be stone free after one treatment and treatment was only unsuccessful in 12.8% of patients (9). This is in comparison to Philipou et al., that also reviewed outcomes for SWL in those older than 70 years. SFR was 63.5% with 23 patients requiring URS, 14 PCNL, one patient required a laparoscopic ureterolithotomy, and 12 patients being either a poor candidate for surgical intervention or declined treatment (8).

PCNL, of all three methods of stone management in the elderly, has been the most researched, however quality evidence in this area remains lacking. Morganstern et al. demonstrated that the elderly population (>80 years) were significantly more likely to have pre operative nitrite positive urine, positive or contaminated urine cultures requiring pre-operative antibiotics, and have a history of urosepsis (28). Indeed, age has been demonstrated to be an independent risk factor for increased levels of bacteriuria when managing larger stones in SWL (29). However, there was no difference between the elderly group and the younger group (21-64 years) for post-operative complications, and length of stay was comparable (30).

Similarly, on comparison of minimally invasive PCNL (mPCNL) and URS for elderly, while mPCNL was more effective for multiple stones, URS was involved with lower complications and post-operative stay (12). Nakamon et al. demonstrated that the elderly population (>65 years) was more likely to be of a higher ASA grade than the younger population, being ASA-1, 1.6% vs. 36.8%; ASA-2, 86.9% vs. 58.7%; and ASA-3, 11.5% vs. 4.4% respectively. However, SFR remained similar between the two groups, and only stone size and previous surgery were found to affect the success rate in a multivariate analysis (30). Indeed, SFR for the elderly population has been reported between 70.5 - 92.8% for PCNL. Resorlu et al. reviewed the impact of increasing comorbidity in the elderly on complication rate and found post operative medical complications were significantly higher in those with >2 comorbidities on the Charlson Comorbidity Index (31).

### Limitations and areas of future research

A review of literature conducted showed few other studies that reported on the outcomes of URS in elderly (10-14). Although the results were similar, elderly were defined being over 60 to 75 years in these studies. The definition of elderly was variable largely (in the current literature it ranged between 60-80 years) due to the age at retirement and the life expectancy in individual countries/regions across the world. With a lack of defined cut-off for elderly, the data remains heterogeneous and reporting and comparison of results is not achievable.

Further research is also required for URS in the elderly, particularly to provide matched analysis to a younger cohort to allow univariate analysis of age on outcome following URS. There is also a lack of the definition of SFR (32). Stone disease in complex patients is rising and requires a tailored approach (20, 21, 33). With a growth in the incidence of stone disease in elderly, future URS studies should focus on outcome measures, which is standardised and these should be carried out in a multi-institutional manner especially comparing it with other treatment modalities.

## CONCLUSIONS

Ureteroscopy for stone disease in elderly is a relatively safe procedure even for large and/or multiple stones with a small risk of minor complications. Cost-effectiveness is demonstrated through the overall length of stay, however prior consideration to social circumstances and pre planning for discharge may negate increased length of stay for those patients who are medically fit for discharge.

## ETHICAL APPROVAL

The local ethics committee approved the study (audit number - 5400). All procedures performed in studies involving human participants were in accordance with the ethical standards of the institutional and/or national research committee and with the 1964 Helsinki declaration and its later amendments or comparable ethical standards.

All patients had given their permission for participating in the study and informed consent was obtained.
